# Intelectin 1 suppresses tumor progression and is associated with improved survival in gastric cancer

**DOI:** 10.18632/oncotarget.3753

**Published:** 2015-04-20

**Authors:** Dan Li, Xiang Zhao, Yong Xiao, Hong Mei, Jiarui Pu, Xuan Xiang, Wanju Jiao, Huajie Song, Hongxia Qu, Kai Huang, Liduan Zheng, Qiangsong Tong

**Affiliations:** ^1^ Department of Surgery, Union Hospital, Tongji Medical College, Huazhong University of Science and Technology, Wuhan, P. R. China; ^2^ Department of Pathology, Union Hospital, Tongji Medical College, Huazhong University of Science and Technology, Wuhan, P. R. China; ^3^ Clinical Center of Human Genomic Research, Union Hospital, Tongji Medical College, Huazhong University of Science and Technology, Wuhan, P. R. China; ^4^ Department of Cardiology, Union Hospital, Tongji Medical College, Huazhong University of Science and Technology, Wuhan, P. R. China

**Keywords:** gastric cancer, intelectin 1, hepatocyte nuclear factor 4 alpha, nuclear factor-kappa B

## Abstract

Recent evidence shows the emerging roles of intelectin 1 (ITLN1), a secretory lectin, in human cancers. Our previous studies have implicated the potential roles of ITLN1 in the aggressiveness of gastric cancer. Herein, we investigated the functions, downstream targets, and clinical significance of ITLN1 in the progression of gastric cancer. We demonstrated that ITLN1 increased the levels of hepatocyte nuclear factor 4 alpha (HNF4α), resulting in suppression of nuclear translocation and transcriptional activity of β-catenin in gastric cancer cells. Mechanistically, ITLN1 attenuated the activity of nuclear factor-kappa B, a transcription factor repressing the HNF4α expression, in gastric cancer cells through inactivating the phosphoinositide 3-kinase/AKT/Ikappa B kinase signaling. Gain- and loss-of-function studies demonstrated that ITLN1 suppressed the growth, invasion, and metastasis of gastric cancer cells *in vitro* and *in vivo*. In addition, restoration of HNF4α expression prevented the gastric cancer cells from ITLN1-mediated changes in these biological features. In clinical gastric cancer tissues, HNF4α expression was positively correlated with that of ITLN1. Patients with high ITLN1 or HNF4α expression had greater survival probability. Taken together, these data indicate that ITLN1 suppresses the progression of gastric cancer through up-regulation of HNF4α, and is associated with improved survival in patients with gastric cancer.

## INTRODUCTION

Gastric cancer is one of the most common cancers worldwide, with approximately 1 million new cases being diagnosed annually [1]. In Asia and parts of South America, gastric cancer is the leading cause of cancer-related death, with a 5-year survival rate below 30% [1]. Better elucidating the mechanisms of tumorigenesis and aggressiveness is important for improving the therapeutic efficiency of gastric cancer [2]. Recent studies have shown that galectins, a family of mammalian lectins that bind β-galactoside, are frequently over-expressed in many human solid and blood malignancies, and exert important functions in tumor biology, including the transformation, growth, adhesion, invasion, and metastasis of cancer cells [3]. Inhibition of galectin-1 expression using an antisense RNA abolishes the membrane anchorage of oncogenic Harvey rat sarcoma viral oncogene homolog (HRAS), and inhibits the HRAS-induced transformation [4]. Galectin-1 also promotes the adhesion of ovarian and prostate cancer cells to extracellular matrix [5, 6], while galectin-3 significantly suppresses the growth and metastasis of breast cancer cells [7]. Galectin-8 reduces the migration of colon cancer cells, indicating its suppressive roles in tumor aggressiveness [8]. Therefore, it is necessary to investigate the roles of lectins in the progression of gastric cancer.

Intelectin 1 (ITLN1), a recently identified secretory and galactofuranose-binding lectin, is mainly expressed in the gastrointestinal goblet cells and omentum, and occasionally in the thymus, bronchus, heart, liver, kidney collecting tubule cells, bladder umbrella and mesothelial cells [9, 10]. Subsequent studies have demonstrated that ITLN1 is involved in the gut immune defense against microorganisms [9, 11–13], insulin-stimulated glucose uptake [14], chronic obstructive pulmonary disease [15], and asthma [16]. Importantly, recent evidence shows that ITLN1 is over-expressed in human malignant pleural mesothelioma (MPM) and secreted into pleural effusions, and serves as a biomarker for distinguishing MPM from lung cancer [17, 18], implicating the emerging roles of ITLN1 in human cancers.

Our previous studies have shown that ITLN1 is aberrantly expressed in gastric cancer, and is a potential prognostic factor for predicting the outcome of gastric cancer patients [19]. However, the exact functions, downstream targets, and clinical significance of ITLN1 in gastric cancer still remain elusive. In the current study, we demonstrate, for the first time, that secretory ITLN1 suppresses the growth, invasion, and metastasis of gastric cancer cells *in vitro* and *in vivo* through up-regulating hepatocyte nuclear factor 4 alpha (HNF4α), and is associated with improved survival of gastric cancer patients. In addition, the activity of nuclear factor-kappa B (NFκB), a transcription factor repressing the expression of HNF4α, is attenuated by ITLN1 via inactivation of phosphoinositide 3-kinase (PI3K)/AKT/Ikappa B kinase (IKK) signaling, suggesting the tumor suppressive roles of ITLN1 in gastric cancer.

## RESULTS

### ITLN1 facilitated the expression of HNF4α at transcriptional levels in gastric cancer cells

**Figure 1:**
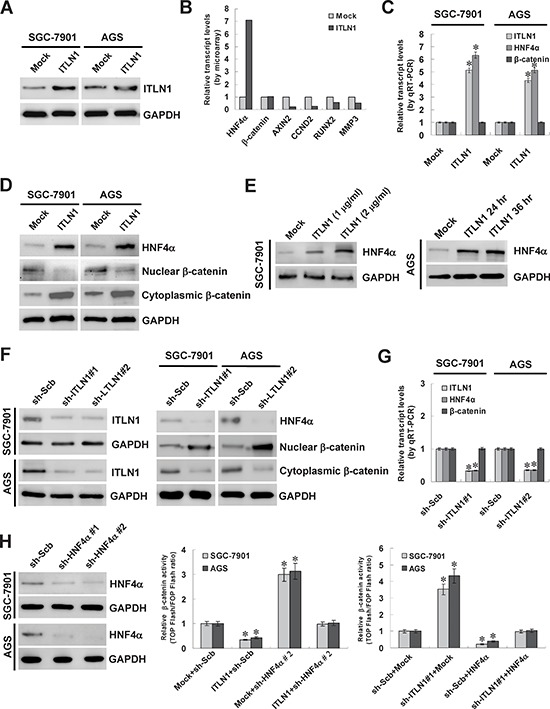
ITLN1 facilitated the expression of HNF4α in gastric cancer cells **A.** *ITLN1* expression vector was stably transfected into cultured gastric cancer cell lines, SGC-7901 and AGS, resulting in increased secretion of ITLN1 into culture supernatant. **B.** microarray analysis revealed the up-regulation of HNF4α in SGC-7901 cells stably transfected with *ITLN1*. **C and D.** real-time quantitative RT-PCR and western blot further demonstrated that stable transfection of *ITLN1* into SGC-7901 and AGS cells increased the transcript and protein levels of HNF4α, when compared to those stably transfected with empty vector (mock). In addition, the nuclear translocation of β-catenin was also decreased in ITLN1 over-expressing SGC-7901 and AGS cells than those in mock cells (*P < 0.01 vs. mock). **E.** recombinant ITLN1 protein (1 and 2 μg/ml) induced the HNF4α expression in SGC-7901 cells at 24 hrs post-administration, than those treated with solvent control (mock). Administration of ITLN1 protein (1 μg/ml) into AGS cells for 24 and 36 hrs resulted in enhanced HNF4α expression than that of mock cells. **F and G.** western blot and real-time quantitative RT-PCR indicated that stable transfection of *ITLN1* shRNA obviously reduced the ITLN1 secretion and down-regulated the HNF4α expression of SGC-7901 and AGS cells, than those of scramble shRNA (sh-Scb)-transfected cells. Moreover, the nuclear translocation of β-catenin was also increased in SGC-7901 and AGS cells transfected with *ITLN1* shRNA, when compared with those transfected with sh-Scb (*P < 0.01 vs. sh-Scb). **H.** western blot and dual-luciferase assays indicated that transfection of shRNA specific for *HNF4α* resulted in its down-regulation in SGC-7901 and AGS cells. Stable over-expression or knockdown of ITLN1 decreased and increased the β-catenin activity in gastric cancer cells, respectively, which was abolished by knockdown or over-expression of HNF4α (*P < 0.01 vs. mock or sh-Scb).

To investigate the potential roles of ITLN1 in gastric cancer, *ITLN1* vector was constructed and stably transfected into cultured gastric cancer cell lines, SGC-7901 and AGS, resulting in enhanced ITLN1 secretion into culture supernatant (Figure 1A). Microarray and gene ontology analyses revealed that stable transfection of *ITLN1* into SGC-7901 cells resulted in altered transcript levels of 1592 human genes, including 547 up-regulated and 1045 down-regulated ones, and regulation of cellular process was the top-ranked function of ITLN1 in gastric cancer cells (Figure S1). Notably, among 331 ITLN1-altered (91 up-regulated and 240 down-regulated) genes involved in the cellular process (Table S1), *HNF4α* was identified as the most up-regulated one (Figure 1B). Real-time quantitative RT-PCR and western blot further demonstrated that stable transfection of *ITLN1* increased the transcript and protein levels of HNF4α, when compared to those stably transfected with empty vector (mock) (Figure 1C and Figure 1D). In addition, administration of recombinant ITLN1 protein (1 and 2 μg/ml) into cultured gastric cancer cell lines also markedly induced the HNF4α expression at 24 and 36 hrs post-administration (Figure 1E). Moreover, the nuclear translocation of β-catenin, a target gene of HNF4α [20] (Figure S2A and Figure S2B), was correspondingly decreased in SGC-7901 and AGS cells stably transfected with *ITLN1* than mock cells (Figure 1C, Figure 1D, and Figure S2C). To further examine the effects of ITLN1 on HNF4α expression, we performed the ITLN1 knockdown experiments. Stable transfection of short hairpin RNA (shRNA) specific for *ITLN1* (sh-ITLN1) led to reduced ITLN1 secretion (Figure 1F, Figure 1G, and Figure S3A), decreased HNF4α expression (Figure 1F, Figure 1G, and Figure S3B), and increased nuclear translocation of β-catenin (Figure 1F and Figure S2D). Moreover, stable over-expression or knockdown of ITLN1 in gastric cancer cells decreased and increased the β-catenin activity and transcription of downstream genes axin 2 (*AXIN2*) [21], cyclin D2 (*CCND2*) [22], runt-related transcription factor 2 (*RUNX2*) [23], and matrix metallopeptidase 3 (*MMP3*) [24], respectively, which was abolished by knockdown or ectopic expression of HNF4α (Figure 1H, Figure S2E, Figure S2F, and Figure S3B). Overall, these results demonstrated that ITLN1 considerably facilitated the HNF4α expression at transcriptional levels in gastric cancer cells.

### Crucial roles of NFκB in ITLN1-mediated regulation of HNF4α in gastric cancer cells

**Figure 2:**
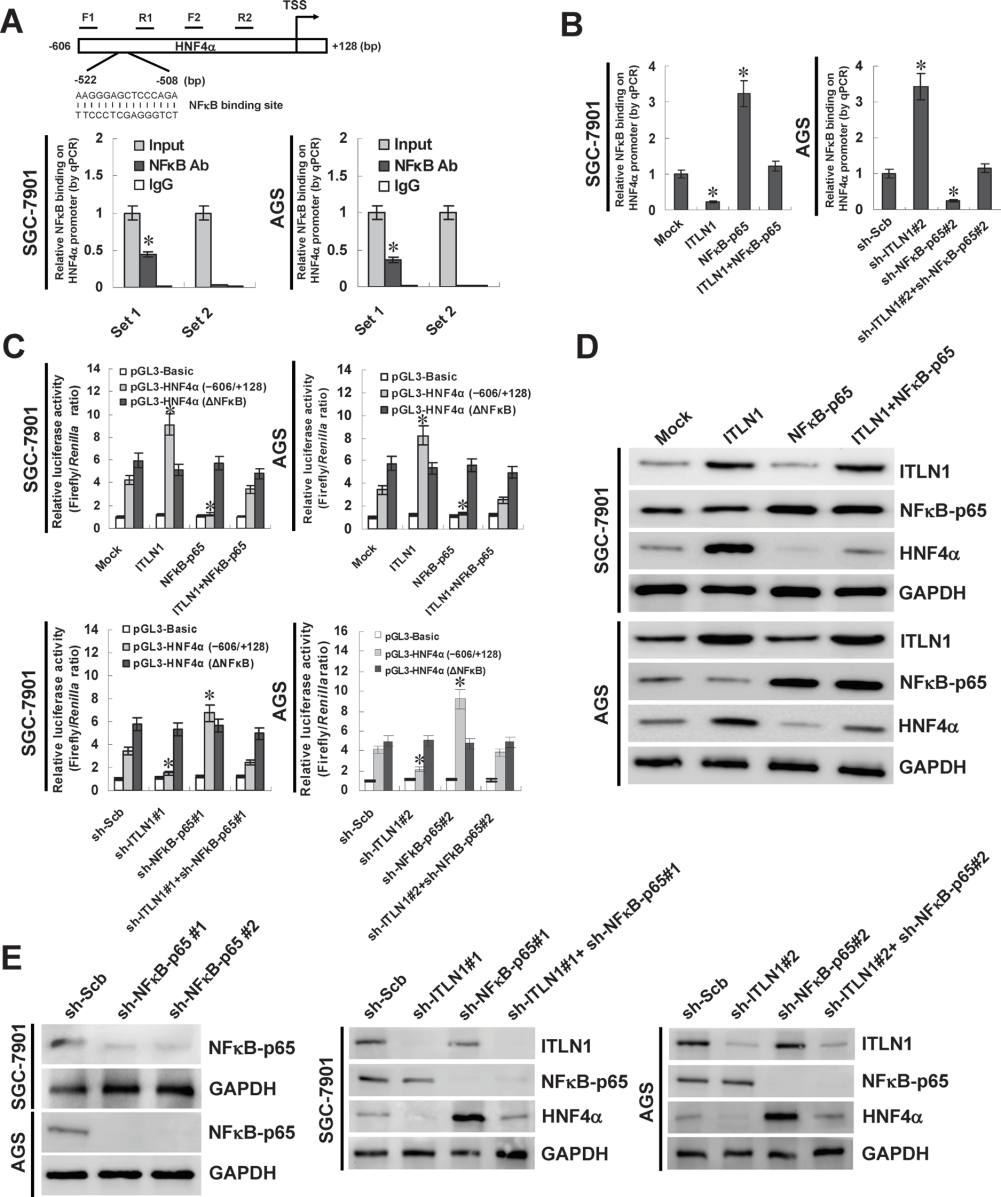
Crucial roles of NFκB in ITLN1-mediated regulation of HNF4α expression in gastric cancer cells **A.** one potential binding site of NFκB was noted within the *HNF4α* promoter, locating at bases 508-522 upstream the transcription start site (TSS). ChIP and qPCR assays indicated the endogenous binding of NFκB-p65 on −606/−409 bp region, but not on −339/−158 region, of the *HNF4α* promoter in SGC-7901 and AGS cells. **B.** ChIP and qPCR assays indicated that over-expression or knockdown of ITLN1 decreased and increased the binding of NFκB-p65 on the *HNF4α* promoter, which was rescued by over-expression and knockdown of NFκB-p65 (*P < 0.01 vs. mock or sh-Scb). **C.** dual-luciferase assay indicated that stable transfection of *ITLN1* or sh-ITLN1 into gastric cancer cells facilitated or suppressed the promoter activity of *HNF4α* than those in mock or sh-Scb-transfected cells, respectively, which was rescued by transfection of *NFκB-p65* or sh-NFκB-p65. In addition, mutation of NFκB binding site abolished these effects (*P < 0.01 vs. sh-Scb or mock). **D.** western blot assay indicated that transfection of *NFκB-p65* prevented the gastric cancer cells from ITLN1-mediated up-regulation of HNF4α. **E.** western blot assay indicated that knockdown of NFκB-p65 prevented gastric cancer cells from sh-ITLN1- repressed expression of HNF4α.

To investigate the mechanisms underlying ITLN1-mediated up-regulation of HNF4α, we analyzed the transcription factor binding sites within the *HNF4α* promoter, and noted one potential binding site of NFκB, locating at bases 508–522 upstream the transcription start site (TSS) (Figure 2A). Chromatin immunoprecipitation (ChIP) and quantitative PCR (qPCR) assays indicated that the endogenous NFκB-targeting *HNF4α* promoter regions were immunoprecipitated using antibody specific for NFκB p65 subunit (NFκB-p65) in cultured SGC-7901 and AGS cells, which were 0.44- and 0.36-fold by normalizing to nuclear extract (input; Figure 2A). As controls, no *HNF4α* promoter regions were immunoprecipitated with unspecific antibody (isotype IgG) or detected by qPCR with primer set (−339/−158 bp) distal to the binding site of NFκB (Figure 2A). In addition, over-expression or knockdown of ITLN1 in SGC-7901 and AGS cells decreased and increased the binding of NFκB-p65 on −606/−409 bp region of the *HNF4α* promoter, which was rescued by over-expression and knockdown of NFκB-p65, respectively (Figure 2B). In addition, dual-luciferase assay indicated that ITLN1 facilitated the *HNF4α* promoter activity, and ectopic expression or knockdown of NFκB-p65 restored the changes in *HNF4α* promoter activity induced by stable transfection of *ITLN1* or sh-ITLN1 in gastric cancer cells, while these effects were abolished by mutation of NFκB-p65 binding site (Figure 2C). Importantly, western blot and real-time quantitative RT-PCR indicated that ectopic expression or knockdown of NFκB-p65 decreased and increased the expression of HNF4α, respectively, and prevented the gastric cancer cells from ITLN1-mediated changes in HNF4α expression (Figure 2D, Figure 2E, Figure S3C, Figure S3D, and Figure S3E). These results indicated that NFκB repressed the transcription of *HNF4α*, and ITLN1 attenuated the function of NFκB to up-regulate HNF4α in gastric cancer cells.

### ITLN1 attenuated the NFκB activity in gastric cancer cells via inactivation of PI3K/AKT/IKK signaling

**Figure 3:**
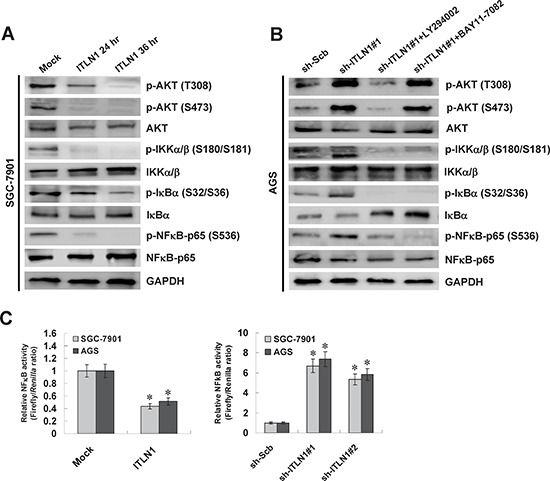
ITLN1 attenuated the NFκB activity via inactivation of PI3K/AKT/IKK signaling in gastric cancer cells **A.** western blot assay indicated that administration of recombinant ITLN1 protein (1 μg/ml for 24 and 36 hrs) reduced the phosphorylation of AKT (T308 and S473), IKKα/β (S180/S181), IκBα (S32/S36), and NFκB-p65 (S536) in SGC-7901 cells, than those treated with solvent control (mock). **B.** western blot assay indicated that stable transfection of *ITLN1* shRNA induced the phosphorylation of AKT (T308 and S473), IKKα/β (S180/S181), IκBα (S32/S36), and NFκB-p65 (S536) in gastric cancer AGS cells, than those of sh-Scb-transfected cells, which were abolished by administration of PI3K activity inhibitor LY294002 (10 μmol/L) and IKK inhibitor BAY11-7082 (50 mmol/L). **C.** dual-luciferase assay indicated that ectopic expression or knockdown of ITLN1 decreased and increased the NFκB activity in SGC-7901 and AGS cells, than those transfected with mock or sh-Scb (*P < 0.01 vs. mock or sh-Scb).

To further explore the roles of NFκB in ITLN1-mediated regulation of HNF4α, we observed the changes in PI3K/AKT/IKK signaling that regulates the activity of NFκB [25]. Administration of recombinant ITLN1 protein (1 μg/ml) into SGC-7901 cells resulted in decreased phosphorylation of AKT (T308 and S473), IKKα/β (S180/S181), Ikappa B alpha (IκBα, S32/S36), and NFκB-p65 (S536) at 24 and 36 hrs, than those treated with solvent control (mock) (Figure 3A). In contrast, stable knockdown of ITLN1 induced the phosphorylation of AKT (T308 and S473), IKKα/β (S180/S181), IκBα (S32/S36), and NFκB-p65 (S536) in gastric cancer cells, which were abolished by administration of PI3K activity inhibitor LY294002 and IKK inhibitor BAY11-7082 (Figure 3B). Accordingly, stable over-expression or knockdown of ITLN1 decreased and increased the NFκB activity in SGC-7901 and AGS cells, respectively (Figure 3C). These data indicated that ITLN1 inhibited the NFκB activity through attenuating the PI3K/AKT/IKK signaling in gastric cancer cells.

### Ectopic expression of ITLN1 suppressed the growth, migration, and invasion of gastric cancer cells through up-regulating HNF4α

**Figure 4:**
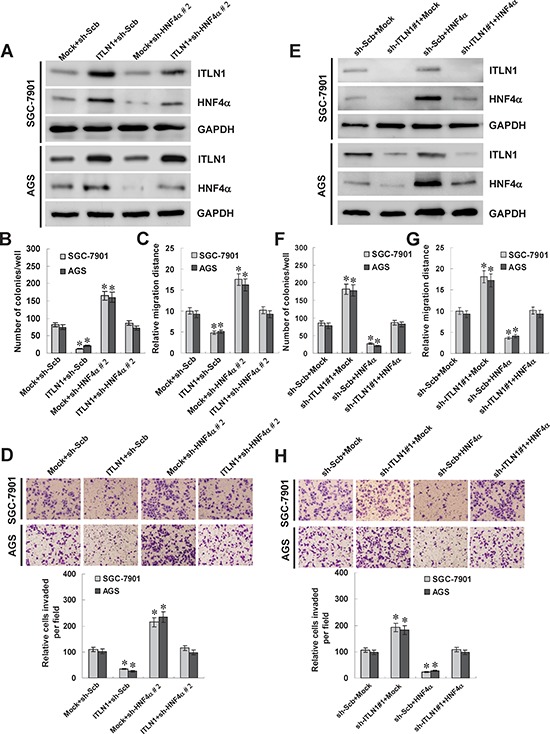
ITLN1 suppressed the growth, migration, and invasion of gastric cancer cells *in vitro* through up-regulating HNF4α **A and E.** western blot indicated that stable transfection of *ITLN1* or sh-ITLN1 into SGC-7901 and AGS cells enhanced and decreased the ITLN1 secretion, respectively, than those transfected with empty vector (mock) or sh-Scb. Transfection of sh-HNF4α or *HNF4α* rescued the ITLN1-mediated changes in HNF4α expression. **B and F.** colony formation assay indicated the decreased or enhanced cell viability of ITLN1 over-expressing or knocking down SGC-7901 and AGS cells. Transfection of sh-HNF4α or *HNF4α* restored the colony formation of ITLN1 over-expressing and knocking down gastric cancer cells, respectively (*P < 0.01 vs. mock or sh-Scb). **C and G.** in scratch assay, the migration of ITLN1 over-expressing or knocking down gastric cancer cells was significantly reduced or increased. Transfection of sh-HNF4α or *HNF4α* rescued the migration of ITLN1 over-expressing and knocking down gastric cancer cells, respectively (*P < 0.01 vs. mock or sh-Scb). **D and H.** matrigel invasion assay indicated the decreased and increased invasion capability of ITLN1 over-expressing or knocking down gastric cancer cells. However, transfection of sh-HNF4α or *HNF4α* restored the invasion of ITLN1 over-expressing and knocking down gastric cancer cells, respectively (*P < 0.01 vs. mock or sh-Scb).

Since previous studies indicate that HNF4α affects the growth, invasion, and metastasis of cancers [26, 27], and combining the evidence that ITLN1 regulated the HNF4α expression, we further investigated the effects of ITLN1 over-expression and target gene restoration on cultured gastric cancer cells. Western blot and real-time quantitative RT-PCR indicated that transfection of shRNA targeting *HNF4α* resulted in its down-regulation, and restored the ITLN1-induced up-regulation of HNF4α in SGC-7901 and AGS cells (Figure 4A and Figure S3F). In colony formation assay, ITLN1 over-expression attenuated the growth of SGC-7901 and AGS cells, when compared to those stably transfected with empty vector (mock) (Figure 4B and Figure S4A). In scratch assay, ITLN1 over-expression attenuated the migration capability of SGC-7901 and AGS cells (Figure 4C and Figure S4B). Matrigel invasion assay showed that gastric cancer cells stably transfected with *ITLN1* presented an impaired invasion capacity than mock cells (Figure 4D). In addition, restoration of HNF4α expression via shRNA transfection rescued the SGC-7901 and AGS cells from their changes in growth, migration, and invasion induced by stable over-expression of ITLN1 (Figure 4B, Figure 4C, Figure 4D, Figure S4A, and Figure S4B). These results revealed the tumor suppressive roles of ITLN1, and indicated that up-regulation of HNF4α was involved in ectopic ITLN1 expression-inhibited aggressiveness of gastric cancer cells.

### Knockdown of ITLN1 promoted the growth, migration, and invasion of gastric cancer cells *in vitro*

To further explore the impacts of ITLN1 on the aggressiveness of gastric cancer cells, we investigated the effects of ITLN1 knockdown and HNF4α restoration on cultured gastric cancer cells. Western blot and real-time quantitative RT-PCR indicated that transfection of *HNF4α* resulted in its over-expression and restored the down-regulation of HNF4α induced by ITLN1 knockdown in SGC-7901 and AGS cells (Figure 4E and Figure S3G). In colony formation assay, knockdown of ITLN1 facilitated the growth of SGC-7901 and AGS cells, when compared to those stably transfected with scramble shRNA (sh-Scb) (Figure 4F and Figure S4C). In scratch assay, ITLN1 knockdown increased the migration capability of SGC-7901 and AGS cells (Figure 4G and Figure S4D). Matrigel invasion assay showed that gastric cancer cells stably transfected with sh-ITLN1 presented an increased invasion capacity than sh-Scb-transfected cells (Figure 4H). In addition, restoration of HNF4α expression via transfection of *HNF4α* vector rescued the SGC-7901 and AGS cells from their changes in growth, migration, and invasion induced by stable knockdown of ITLN1 (Figure 4F, Figure 4G, Figure 4H, Figure S4C, and Figure S4D). These findings suggested that identification of *HNF4α* as an ITLN1 target gene may explain, at least in part, why ITLN1 suppressed the growth, migration, and invasion of gastric cancer cells.

### ITLN1 suppressed the growth and metastasis of gastric cancer cells *in vivo*

**Figure 5:**
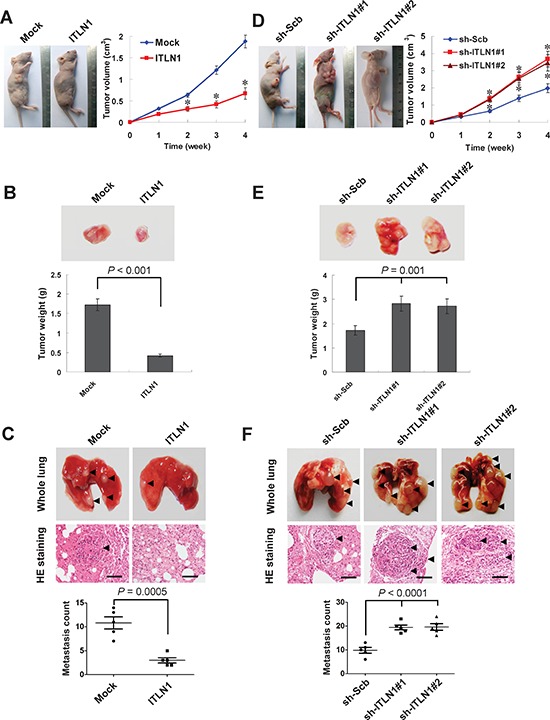
ITLN1 attenuated the growth and metastasis of gastric cancer cells *in vivo* **A and B.** hypodermic injection of SGC-7901 cells into athymic nude mice established subcutaneous xenograft tumors. Four weeks later, mice (*n* = 5) from each group were sacrificed. Stable transfection of *ITLN1* into cancer cells resulted in decreased tumor size (*P < 0.01 vs. mock), and the mean tumor weight formed from ITLN1 over-expressing cells was significantly decreased. **C.** SGC-7901 cells were injected into the tail vein of athymic nude mice (*n* = 5 for each group). Cancer cells stably transfected with *ITLN1* established significantly fewer metastatic colonies (arrowhead). Scale bars: 100 μm. **D and E.** stable transfection of sh-ITLN1 into cancer cells resulted in increased tumor size (*P < 0.01 vs. sh-Scb), and the mean tumor weight formed from ITLN1 knockdown cells was significantly increased. **F.** cancer cells stably transfected with sh-ITLN1 established significantly more metastatic colonies (arrowhead), when compared to those transfected with sh-Scb. Scale bars: 100 μm.

We next investigated the efficacy of ITLN1 against tumor growth and metastasis *in vivo*. Stable transfection of *ITLN1* into SGC-7901 cells resulted in decreased growth and tumor weight of subcutaneous xenograft tumors in athymic nude mice, when compared to those stably transfected with empty vector (mock) (Figure 5A and Figure 5B). In the experimental metastasis studies, SGC-7901 cells stably transfected with *ITLN1* established statistically fewer lung metastatic colonies than mock group (Figure 5C). On the other hand, stable knockdown of ITLN1 in SGC-7901 cells resulted in increased growth and tumor weight of subcutaneous xenograft tumors in athymic nude mice (Figure 5D and Figure 5E), and more lung metastatic colonies (Figure 5F), when compared to those stably transfected with sh-Scb. These results were consistent with the *in vitro* findings that ITLN1 suppressed the growth, migration, and invasion of gastric cancer cells.

### Expression of HNF4α and ITLN1 was positively correlated and associated with improved survival in clinical gastric cancer cases

**Figure 6:**
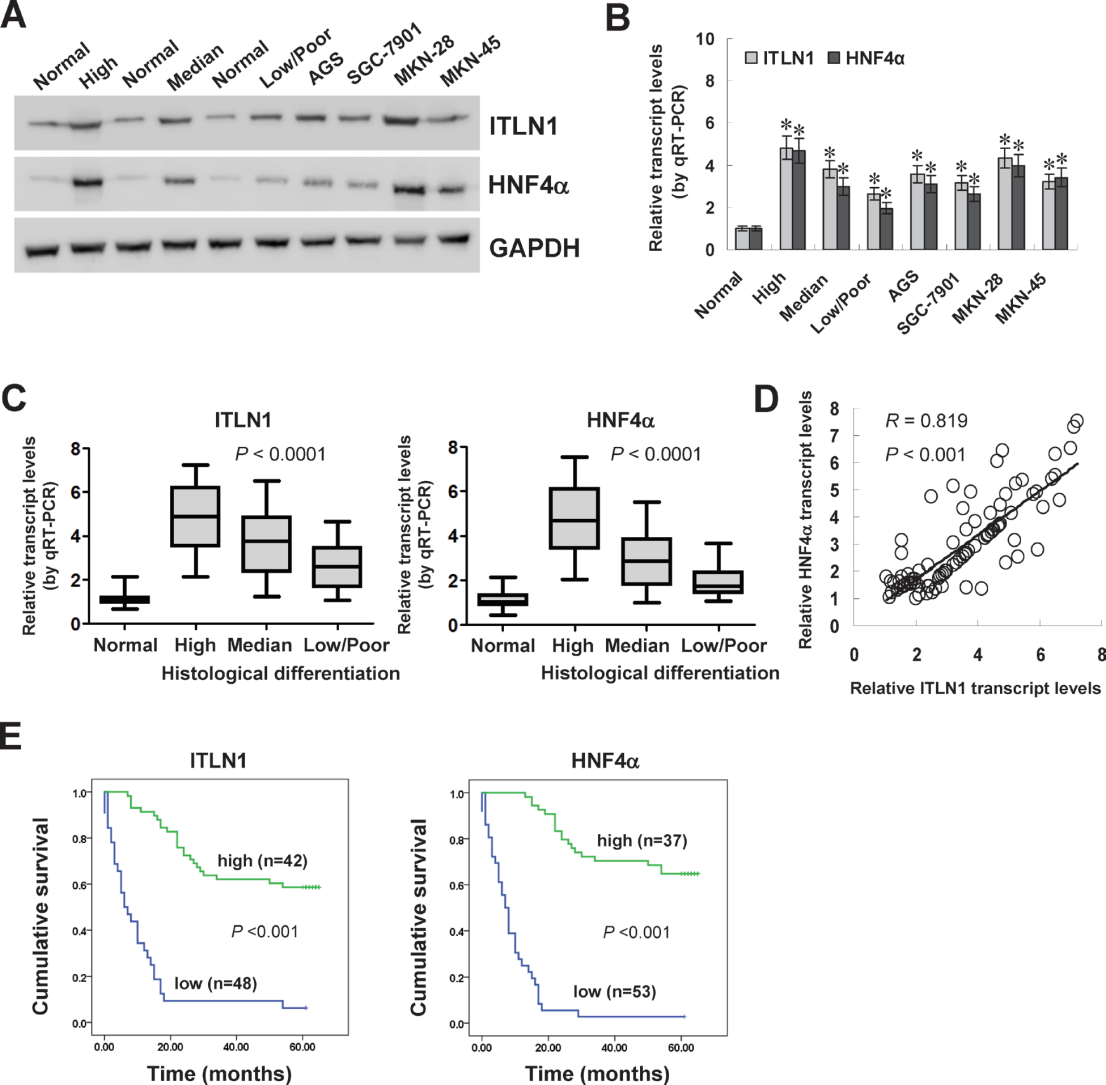
HNF4α was positively correlated with ITLN1 expression in gastric cancer tissues and cell lines **A and B.** western blot and real-time quantitative RT-PCR revealed higher expression levels of ITLN1 and HNF4α in gastric cancer tissues (*n* = 90) and cultured cell lines AGS, SGC-7901, MKN-28, and MKN-45 than those in normal gastric mucosa (*n* = 30; *P < 0.01 vs. normal gastric mucosa). **C.** real-time quantitative RT-PCR indicated that the transcript levels of *ITLN1* and *HNF4α* were gradually decreased in gastric cancer tissues with high (*n* = 22), median (*n* = 30), and low/poor (*n* = 38) differentiation. **D.** there was a positive correlation between *ITLN1* and *HNF4α* transcript levels in gastric cancer tissues (*n* = 90). **E.** Kaplan–Meier survival analysis revealed that patients with high expression of ITLN1 or HNF4α had greater survival probability than those with low expression.

To investigate the expression correlation between ITLN1 and HNF4α in gastric cancer tissues, clinical specimens from 90 primary cases were collected. Western blot and real-time quantitative RT-PCR were applied to measure the expression levels of ITLN1 and HNF4α in gastric cancer specimens, normal gastric mucosa, and cultured cell lines AGS, SGC-7901, MKN-28, and MKN-45. As shown in Figure 6A and Figure 6B, higher protein and transcript levels of ITLN1 or HNF4α were observed in gastric cancer tissues and cell lines than those in normal gastric mucosa, while their expression levels gradually decreased along with the histological differentiation grades of gastric cancer (Figure 6C, *P* < 0.0001), which was in line with the results from public databases (Figure S5A, Figure S5B, and Figure S5C). The ITLN1 levels were significantly lower in gastric cancer cases with poor differentiation (*P* = 0.019), deeper gastric wall invasion (*P* < 0.001), lymph node metastasis (*P* < 0.001), and advanced tumor-node-metastasis (TNM) stage (*P* = 0.001) (Table S2). Notably, there was a positive correlation between *ITLN1* and *HNF4α* transcript levels in gastric cancer tissues (correlation coefficient *R* = 0.819, *P* < 0.001, Figure 6D), which was consistent with the results from public datasets (Figure S5D). Kaplan–Meier survival analysis revealed that patients with high ITLN1 (*P* < 0.001) or HNF4α (*P* < 0.001) expression had greater survival probability than those with low expression (Figure 6E). Taken together, these results demonstrated that the expression of HNF4α and ITLN1 was positively correlated and associated with improved survival in clinical gastric cancer cases.

## DISCUSSION

Since human *ITLN1* was isolated from a small intestine cDNA library in 2001 [28], a series of studies have indicated the potential roles of ITLN1 in the tumorigenesis [17, 19, 29, 30]. Ectopic expression of ITLN1 into prostate cancer cells results in significantly decreased *in vitro* cell viability; meanwhile, increased tumorigenicity and *in vivo* growth are observed in ITLN1 knockdown prostate cancer cells [29]. ITLN1 induces the apoptosis of hepatocellular carcinoma cells through inhibiting p53 deacetylation in a silent mating type information regulation 2 homolog 1-dependent manner [30]. Based on the high ITLN1 levels in colon adenocarcinoma, it is plausible to speculate the involvement of ITLN1 in gastrointestinal malignancies [17]. Our previous studies have shown the aberrant expression of ITLN1 in gastric cancer specimens, which was significantly associated with tumor progression and patients' outcome [19]. In the current study, we further demonstrated that secretory ITLN1 suppressed the growth, migration, invasion, and metastasis of gastric cancer cells *in vitro* and *in vivo*, suggesting the tumor suppressive roles of ITLN1 in gastric cancer.

HNF4α, a member of nuclear receptor superfamily, is a versatile transcription factor that regulates gene expression through binding to cognate DNA sequences [31]. Previous evidence shows that HNF4α expression is diminished in hepatocellular carcinoma [20, 32–34], and loss of HNF4α leads to increased hepatocyte proliferation and promotion of diethylnitrosamine-induced hepatic tumors secondary to aberrant c-Myc activation [26]. In addition, over-expression of HNF4α induces the differentiation of hepatoma cells into more mature phenotypes, and abolishes the tumorigenesis [35]. Down-regulation of HNF4α is also involved in the metastasis and worse prognosis of colorectal cancer [27]. Recent studies show that HNF4α suppresses the β-catenin activity in hepatocytes, through increasing the E-cadherin/β-catenin complex at the plasma membrane and subsequently decreasing the nuclear translocation of β-catenin, resulting in inhibition of epithelial to mesenchymal transition and carcinogenesis [20]. Moreover, HNF4α is expressed in human gastric cancer [36], and absence of HNF4α expression is related to tumor invasion [37]. However, the exact functions of HNF4α in gastric cancer still remain largely unknown. In this study, we demonstrated that HNF4α expression in gastric cancer specimens was associated with patients' survival. In addition, we found that HNF4α inhibited the nuclear translocation and transcription activity of β-catenin, and suppressed the growth and aggressiveness of gastric cancer cells, suggesting the crucial roles of HNF4α in the tumorigenesis and progression of gastric cancer.

Human *HNF4α* gene is regulated mainly at the transcriptional level [38]. The *HNF4α* promoter region contains several binding sites of transcription factors, such as hepatocyte nuclear factor 1 alpha, specificity protein 1, hepatocyte nuclear factor 6 alpha, and GATA binding protein 6, which are crucial for high HNF4α expression in hepatocellular carcinoma and hepatoblastoma cells [38]. Transcription factor Snail represses the expression of HNF4α in hepatocytes through direct binding with the *HNF4α* promoter region [39]. As a transcription repressor, high mobility group A2 suppresses the transcription of *HNF4α* in mammary epithelial cells via transforming growth factor beta 1-induced SMAD family member 3 signaling pathway [40]. In response to cellular stress, including chemotherapeutic drug (doxorubicin) treatment and ultraviolet irradiation, p53 protein is activated to suppress the HNF4α expression via binding with its promoter in the hepatocytes [41]. In this study, our experimental evidence demonstrated that *HNF4α* was a target of transcription factor NFκB in gastric cancer cells. First, ChIP assay indicated the enrichment of NFκB on the *HNF4α* promoter. Second, the activity of *HNF4α* promoter was responsive to over-expression or knockdown of NFκB. Third, mutation of the NFκB binding site abolished its regulatory effects on the *HNF4α* promoter luciferase reporter. Finally, endogenous HNF4α expression, both mRNA and protein, was altered by over-expression and knockdown of NFκB in gastric cancer cells, suggesting that NFκB may regulate the HNF4α expression by repressing its transcription.

It has been indicated that PI3K/AKT/IKK pathway is a major cascade mediating the activation of NFκB in human cancer cells [25]. AKT/IKK signaling phosphorylates the IκBα at Ser32 and Ser36 to facilitate its ubiquitin-related degradation, resulting in release and nuclear translocation of NFκB to regulate gene transcription [42]. In addition, PI3K/AKT may act through IKKα/β to increase the phosphorylation of p65 subunit of NFκB at Ser536 and enhance the NFκB transactivation [42]. Our data showed that ITLN1 suppressed the activity of NFκB through inactivating PI3K/AKT/IKK pathway, resulting in increased HNF4α expression in gastric cancer cells. Moreover, restoration of HNF4α expression rescued the gastric cancer cells from ITLN1-mediated suppressive phenotypes, suggesting that ITLN1 may exert its tumor suppressive function, at least in part, through up-regulation of HNF4α in gastric cancer.

In summary, for the first time, we have demonstrated that secretory ITLN1 efficiently inhibited the growth, invasion, and metastasis of gastric cancer cells *in vitro* and *in vivo* through up-regulating HNF4α in a PI3K/AKT/IKK/NFκB inactivation-dependent manner. Gastric cancer patients with high ITLN1 or HNF4α expression had improved survival probability. This study extends our knowledge about the regulation of tumor suppressive genes associated with the progression of gastric cancer, and suggests that ITLN1 may be of potential values as a novel therapeutic target for gastric cancer.

## MATERIALS AND METHODS

### Patient tissue samples

Approval to conduct this study was obtained from the Institutional Review Board of Tongji Medical College (approval number: 2011-S085). Fresh specimens of 90 well-established primary gastric cancer cases were obtained from the Department of Surgery, Union Hospital of Tongji Medical College. Their pathological diagnosis was proved by at least two pathologists. The demographic and clinicopathological data of all patients were summarized in Table S2. Adjacent gastric mucosa specimens that contained no macroscopic tumor were also obtained, and the non-neoplastic areas were subsequently verified by microscopic histology to be free of tumor infiltration. The fresh tumor and adjacent normal gastric specimens were collected and stored at −80°C until use.

### Cell culture

Human gastric cancer cell lines AGS (CRL-1739), SGC-7901, MKN-28 and MKN-45 were obtained from the American Type Culture Collection (Rockville, MD) and Type Culture Collection of Chinese Academy of Sciences (Shanghai, China). Cell lines were authenticated on the basis of viability, recovery, growth, morphology, and isoenzymology by the provider. Cell lines were used within 6 months after resuscitation of frozen aliquots, and grown in RPMI1640 medium (Life Technologies, Inc., Gaithersburg, MD) supplemented with 10% fetal bovine serum (Life Technologies, Inc.), penicillin (100 U/ml), and streptomycin (100 μg/ml). Cells were maintained at 37°C in a humidified atmosphere of 5% CO_2_. Cells were incubated in serum-free RPMI1640 for 4 hrs, and treated with recombinant ITLN1 protein (Enzo Life Sciences, Farmingdale, NY), LY294002, or BAY 11-7082 (Calbiochem, La Jolla, CA) as indicated.

### Gene over-expression and knockdown

Human *ITLN1* cDNA (942 bp) and *NFκB-p65* cDNA (1656 bp) were amplified from the gastric cancer tissues (Table S3) and subcloned into pcDNA3.1 (Clontech, Mountain View, CA). The oligonucleotides encoding shRNA specific for *ITLN1*, *HNF4α*, and *NFκB-p65* (Table S3) were subcloned into the *Bam H* I and *Hind* III restrictive sites of GV102 (Genechem Co., Ltd, Shanghai, China). The *ITLN1* or *ITLN1* shRNA vectors were transfected into cancer cells with Lipofectamine 2000 (Life Technologies, Inc.), and stable cell lines were screened by administration of neomycin (Invitrogen, Carlsbad, CA). The pcDNA3.1 and sh-Scb were applied as controls (Table S3).

### Rescue of target gene expression

To restore the ITLN1 knockdown-induced down-regulation of HNF4α, stable cell lines were transfected with the *HNF4α* expression vector provided by Dr. David Martinez Selva [43]. To rescue the ITLN1-induced up-regulation of HNF4α, the shRNA specific for *HNF4α* (Table S3) was transfected into cancer cells with Genesilencer Transfection Reagent (Genlantis, San Diego, CA). The sh-Scb was applied as a control (Table S3).

### Whole genome expression microarray and gene ontology analysis

Whole genome expression was determined using high-throughput mRNA microarray analysis following Minimum Information About a Microarray Experiment (MIAME) guidelines. Briefly, total RNA of 1 × 10^6^ cells was isolated using TRIzol® reagent (Life Technologies, Inc.). RNA was tested for purity and DNA contamination using A260/A280 ratio and standard denaturing agarose gel electrophoresis. Gene expression profiling was performed using Agilent Whole Human Genome Oligo Microarray 4 × 44K at Shanghai Technology Corporation (Shanghai, China). The slides were scanned with Agilent Microarray Scanner (Agilent Technologies, Santa Clara, CA). Raw data intensities were extracted from the aligned scanned images and normalized. Raw microarray data were submitted to the Gene Expression Omnibus database (Accession No. GSE58962). Gene ontology analysis was performed using Ingenuity Pathway Analysis (Qiagen, Redwood City, CA).

### Western blot

Tissue or cellular protein was extracted with 1 × cell lysis buffer (Promega, Madison, WI). Culture supernatant was concentrated using a 10,000 MWCO spin column (Millipore, Billerica, MA). Protein expression in lysate or supernatant was analyzed by western blot as previously described [44–48], with antibodies specific for ITLN1, HNF4α (Abcam, Cambridge, MA), β-catenin, phosphorylated AKT (p-AKT, T308), p-AKT (S473), AKT, phosphorylated IKKα/β (p-IKKα/β, S180/S181), IKKα/β, phosphorylated IκBα (p-IκBα, S32/S36), IκBα, phosphorylated NFκB-p65 (p-NFκB-p65, S536), NFκB-p65 (Cell Signaling, Danvers, MA), and glyceraldehyde 3-phosphate dehydrogenase (GAPDH; Santa Cruz Biotechnology, Santa Cruz, CA).

### Real-time quantitative RT-PCR

Total RNA was isolated with RNeasy Mini Kit (Qiagen Inc., Valencia, CA). The reverse transcription reactions were conducted with Transcriptor First Strand cDNA Synthesis Kit (Roche, Indianapolis, IN). The PCR primers for *ITLN1*, *HNF4α*, *β-catenin*, *AXIN2*, *CCND2*, *RUNX2*, *MMP3*, and *GAPDH* were indicated in Table S4. Real-time PCR was performed with SYBR Green PCR Master Mix (Applied Biosystems, Foster City, CA). The fluorescent signals were collected during extension phase, and the Ct values of sample were calculated. The transcript levels were normalized to GAPDH and analyzed by 2^−ΔΔCt^ method. The degree of transcript levels in cancer tissue specimens was classified into “high expression” and “low expression” groups, in which mRNA levels were higher or lower than the median expression in all samples, respectively.

### Luciferase reporter assay

Human *HNF4α* promoter (−606/+128 bp relative to TSS) was amplified from Genomic DNA by PCR (Table S3), and inserted into firefly luciferase reporter vector pGL3-Basic (Promega). The NFκB *cis*-reporter pNFκB-Luc was purchased from Stratagene (La Jolla, CA). The β-catenin activity reporter plasmids, TOP-FLASH and FOP-FLASH, were obtained from Millipore. Dual-luciferase assay was performed as previously described [48, 49]. Relative β-catenin activation was determined by the TOP-FLASH/FOP-FLASH ratio from at least three independent experiments.

### Chromatin immunoprecipitation

ChIP assay was performed according to the manufacture's instructions of EZ-ChIP kit (Upstate Biotechnology, Temacula, CA) [48–50]. DNA was sonicated into fragments of an average size of 200 bp. Different PCR primer sets were designed, distributing adjacent or distal to the NFκB binding site on the *HNF4α* promoter (Table S4). Real-time qPCR with SYBR Green PCR Master Mix was performed using ABI Prism 7700 Sequence Detector. The amount of immunoprecipitated DNA was calculated in reference to a standard curve and normalized to input DNA.

### Nuclear translocation assay

Cancer cells were plated on coverslips, fixed with a solution of 95% ethanol and 5% glacial acetic acid at −20°C for 20 min, permeabilized with 0.3% Triton X-100 in phosphate buffered saline (PBS) at room temperature for 5 min, and blocked with 5% milk for 1 hr. Cells were incubated at 4°C overnight with primary antibody specific for β-catenin (Millipore; 1:100 dilution). After washing with PBS, cells were incubated with Alexa Fluor 594 goat anti-rabbit IgG (1:1000 dilution) at room temperature for 30 min. Cells were stained with 4′, 6-diamidino-2-phenylindole (DAPI, 300 nmol/L in PBS) to visualize nuclei and photographed with a Nikon Eclipse E800 microscope.

### Colony formation assay

Cancer cells were seeded at a density of 300 cells/ml on 35-mm dishes. Colony formation assay was performed as previously described [45]. Positive colony formation (more than 50 cells/colony) was counted. The survival fraction of cells was expressed as the ratio of plating efficiency of treated cells to that of untreated control cells.

### Scratch migration assay

Cancer cells were cultured in 24-well plates and scraped with the fine end of 1-ml pipette tips (time 0). Plates were washed twice with PBS to remove the detached cells, and incubated with the complete growth medium. Cell migration was photographed using 10 high-power fields, at 0, 24 hr post-induction of injury. Remodeling was measured as diminishing distance across the induced injury, normalized to the 0 hr control, and expressed as outgrowth (μm) [50].

### Matrigel invasion assay

Matrigel invasion assay was performed using membranes coated with Matrigel matrix (BD Science, Sparks, MD). Homogeneous single cell suspensions (1 × 10^5^ cells/well) were added to the upper chambers and allowed to invade for 24 hrs at 37°C in a CO_2_ incubator. Invaded cells were stained with 0.1% crystal violet for 10 min at room temperature and examined by light microscopy. Quantification of invaded cells was performed according to published criteria [44, 45].

### *In vivo* growth and metastasis assay

All animal experiments followed the national guidelines for the care and use of animals, and were approved by the Animal Care Committee of Tongji Medical College (approval number: Y20080290). For the *in vivo* tumor growth studies, 2-month-old male nude mice (*n* = 5 per group) were injected subcutaneously in the upper back with 1 × 10^6^ cancer cells. One month later, mice were sacrificed and examined for tumor weight. The experimental metastasis (0.4 × 10^6^ cancer cells per mouse, *n* = 5 per group) studies were performed with 2-month-old male nude mice as previously described [44, 45].

### Statistical analysis

Unless otherwise stated, all data were shown as mean ± standard error of the mean. The SPSS 18.0 statistical software (SPSS Inc., Chicago, IL) was applied for statistical analysis. The χ^2^ analysis and Fisher exact probability analysis were applied for comparison among the expression of ITLN1, HNF4α, and individual clinicopathological features. Pearson's coefficient correlation was applied for analyzing the relationship between ITLN1 and HNF4α expression. The Kaplan-Meier method was used to estimate the survival rates, and the log-rank test was used to assess the survival difference. Difference of cancer cells was determined by *t* test or analysis of variance (ANOVA).

## SUPPLEMENTARY FIGURES AND TABLES


