# Supplementary material for: Intelectin 1 suppresses tumor progression and is associated with improved survival in gastric cancer
Source: Oncotarget. 2015 Apr 20;6(18):16168–82. doi: 10.18632/oncotarget.3753 (PMC4599263; doi:10.18632/oncotarget.3753)
Supplement: Supplementary file 1 [file oncotarget-06-16168-s001.pdf]

SUPPLEMENTARY FIGURES AND TABLES

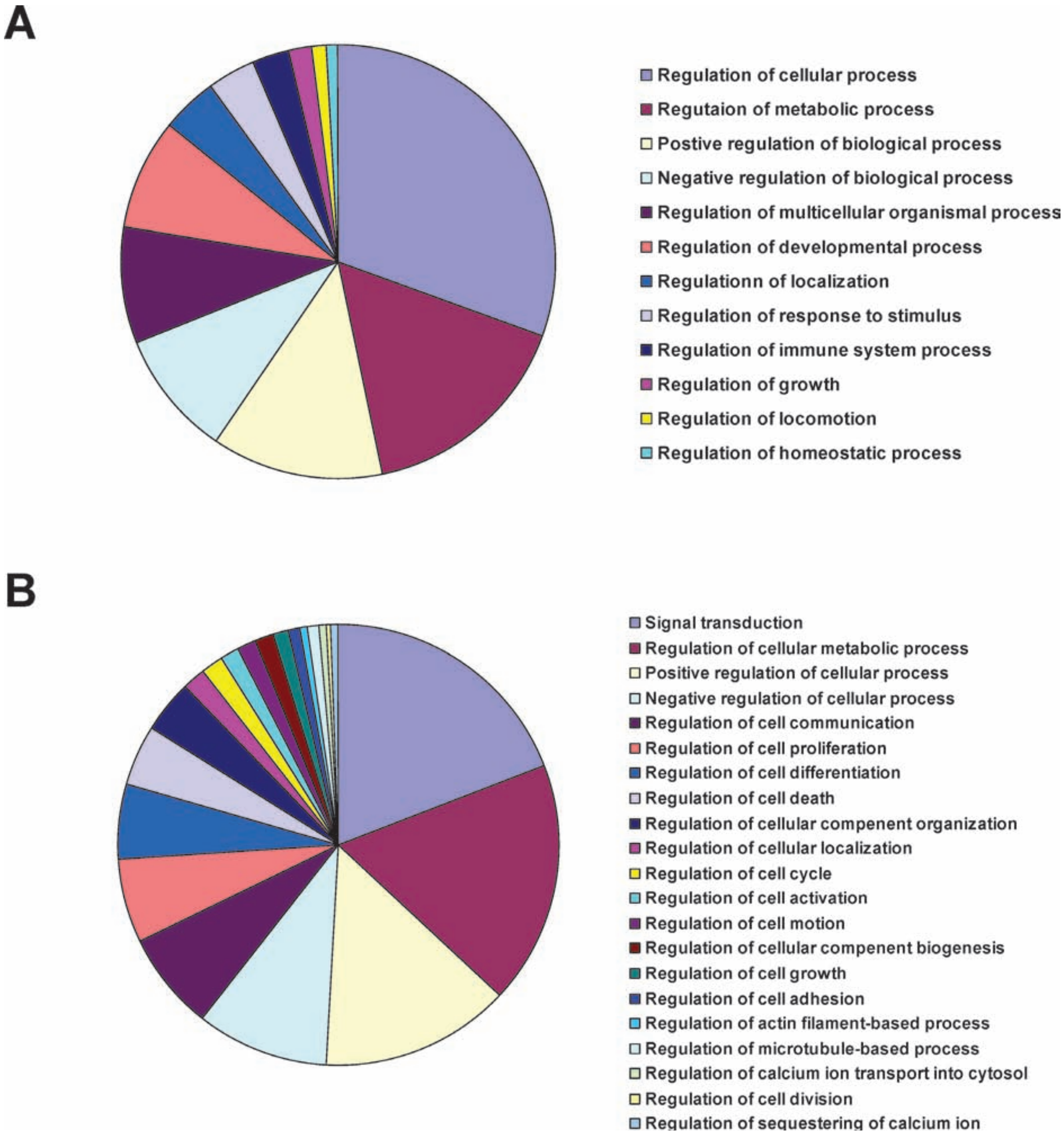

Supplementary Figure S1: Gene ontology analysis. Gene ontology analysis of microarray data **A.** revealed that regulation of cellular process **B.** was the top-ranked function of ITLN1 in gastric cancer SGC-7901 cells stably transfected with *ITLN1*.

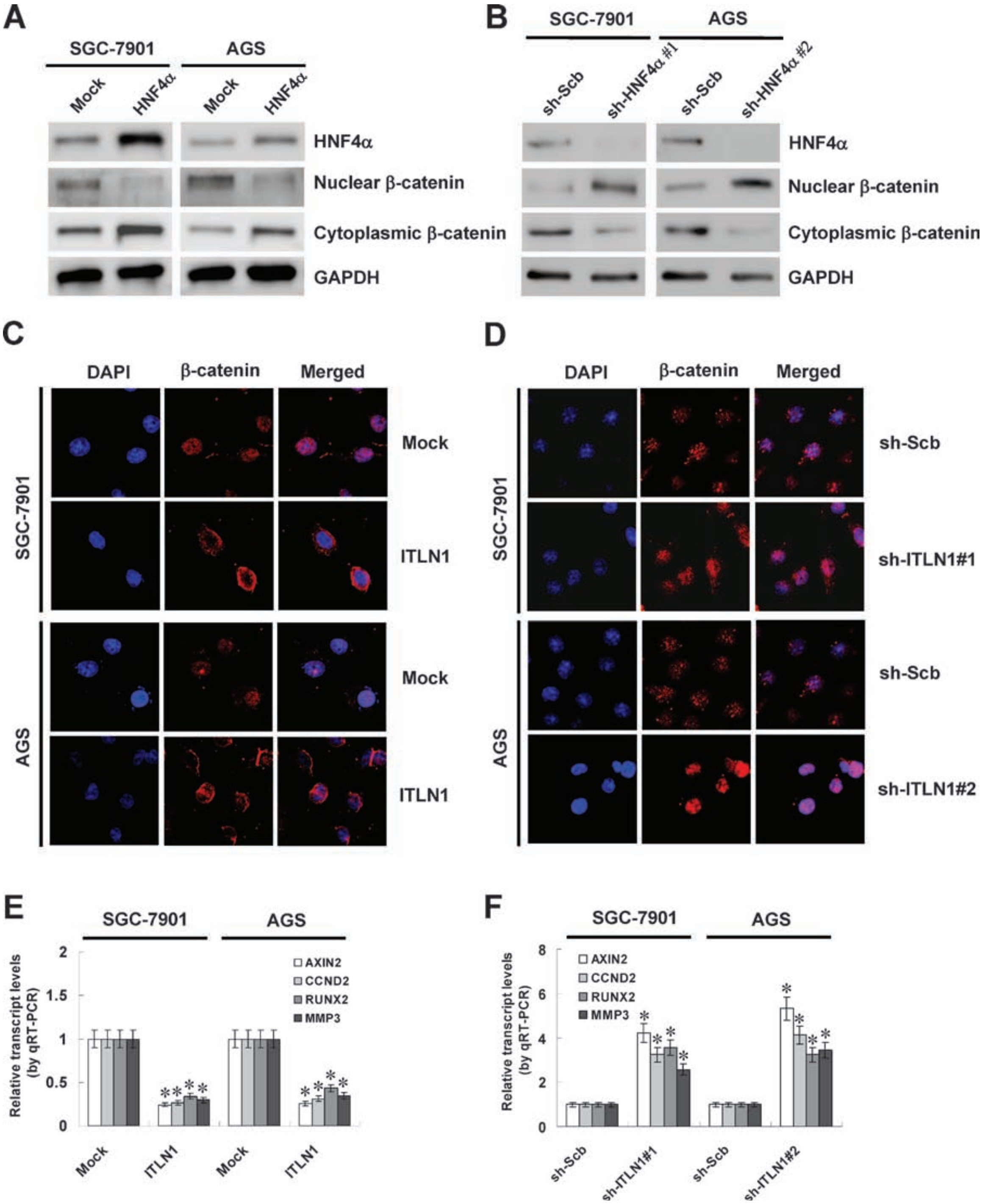

Supplementary Figure S2: Effects of HNF4α and ITLN1 on nuclear translocation and activation of β-catenin in gastric cancer cells. A and B. western blot indicated that transfection of *HNF4α* or sh-HNF4α into SGC-7901 and AGS cells resulted in decreased and increased nuclear translocation of β-catenin. **C and D.** confocal observation indicated that transfection of *ITLN1* or sh-ITLN1 into SGC-7901 and AGS cells resulted in decreased and increased nuclear translocation of β-catenin, than those transfected with mock or sh-Scb. **E and F.** real-time quantitative RT-PCR assay indicated that transfection of *ITLN1* or sh-ITLN1 into SGC-7901 and AGS cells decreased or increased the transcription of β-catenin downstream genes (*AXIN2*, *CCND2*, *RUNX2*, and *MMP3*), than those transfected with mock or sh-Scb (**P* < 0.01 vs. mock or sh-Scb).

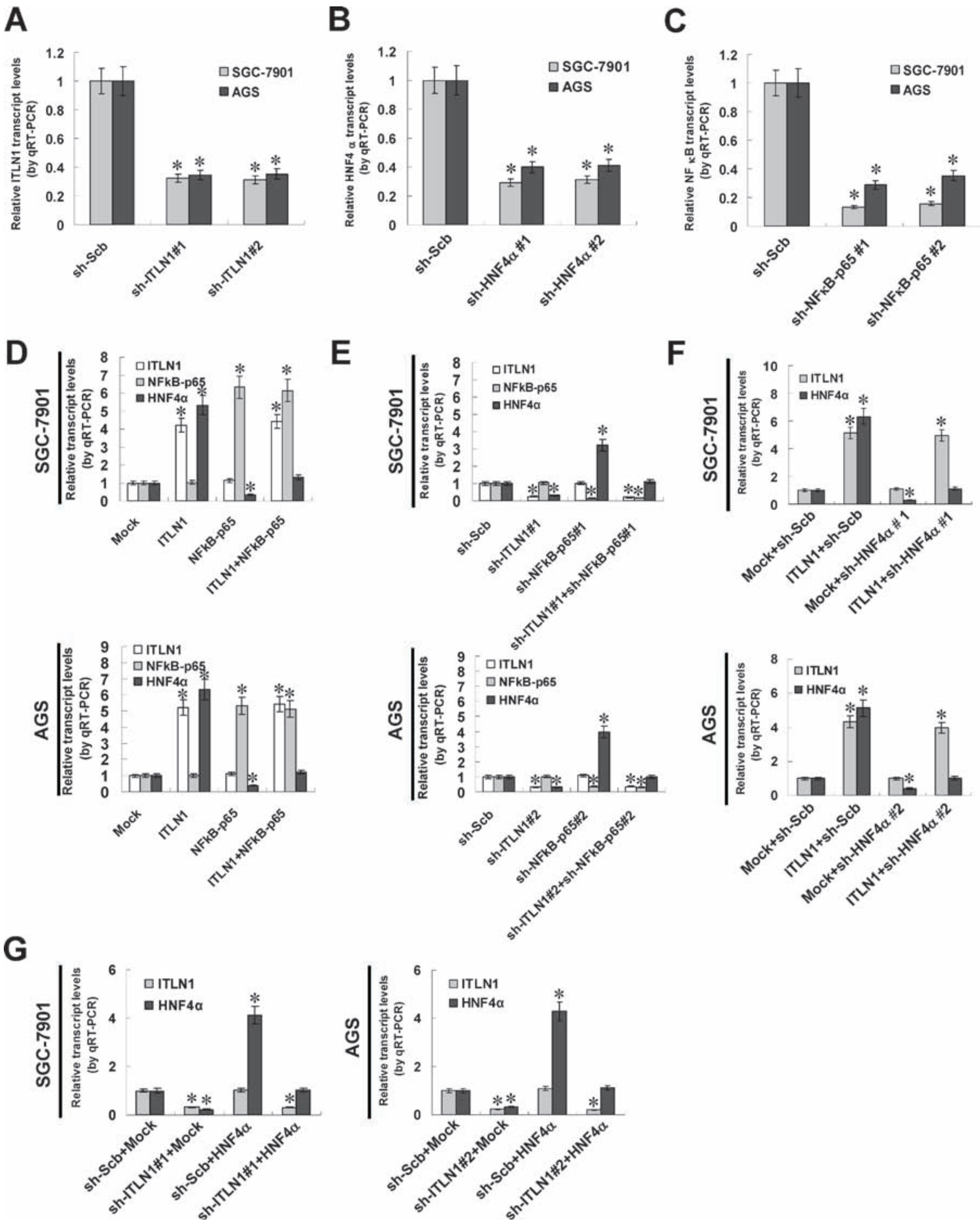

Supplementary Figure S3: Regulatory roles of ITLN1 and NFκB in HNF4α expression. A, B, and C. real-time quantitative RT-PCR indicated that transfection of shRNAs specific for *ITLN1*, *HNF4α* and *NFκB-p65* resulted in their down-regulation in gastric cancer SGC07901 and AGS cells, than those transfected with sh-Scb (**P* < 0.01 vs. sh-Scb). **D and E.** real-time quantitative RT-PCR indicated that over-expression or knockdown of NFκB-p65 decreased and increased the expression of HNF4α, respectively, and prevented the gastric cancer SGC07901 and AGS cells from ITLN1-mediated changes in HNF4α expression, than those transfected with empty vector (mock) or sh-Scb (**P* < 0.01 vs. mock or sh-Scb). **F and G.** real-time quantitative RT-PCR indicated that transfection of sh-HNF4α or *HNF4α* decreased and increased the expression of HNF4α, respectively, and prevented the gastric cancer SGC07901 and AGS cells from ITLN1-mediated changes in HNF4α expression, than those transfected with sh-Scb or mock (**P* < 0.01 vs. mock+sh-Scb).

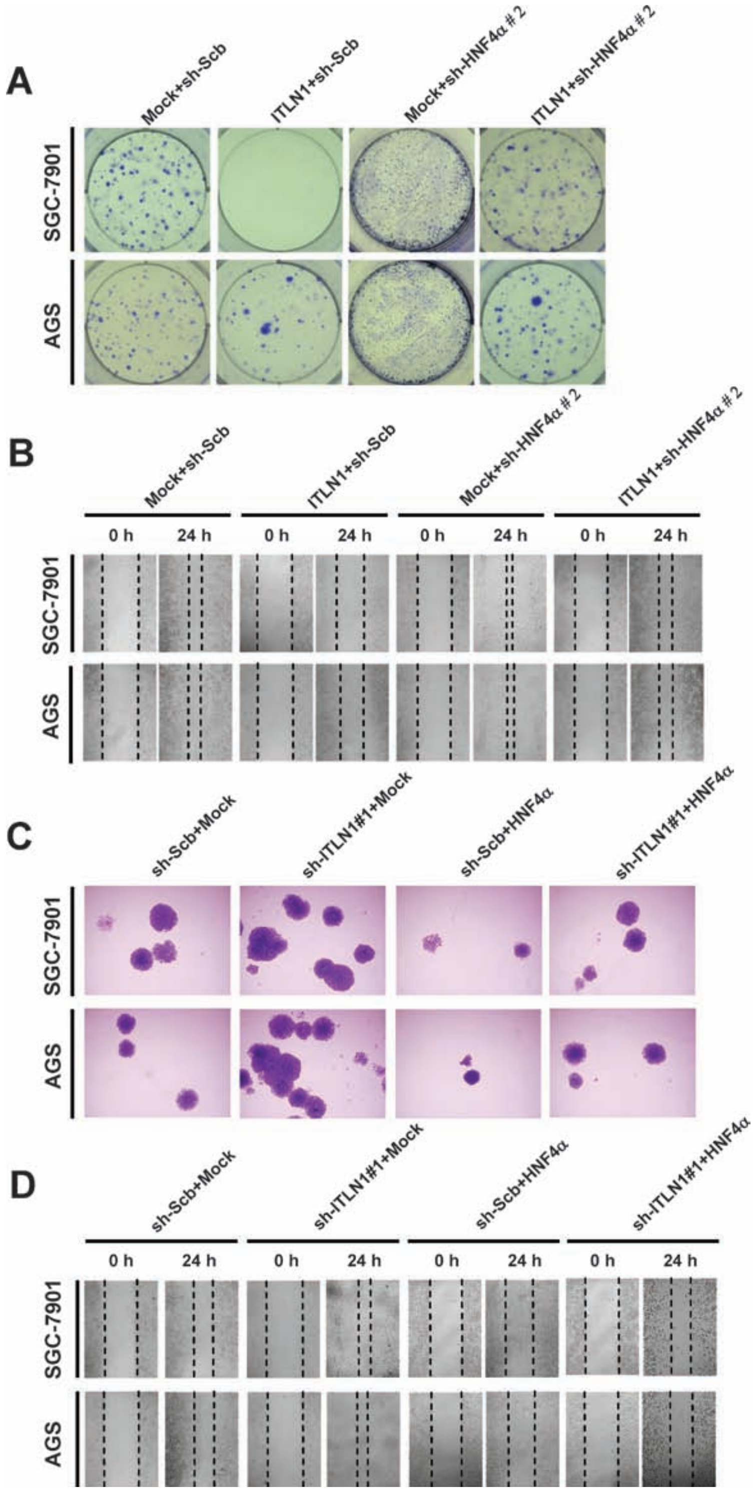

Supplementary Figure S4: ITLN1 suppressed the aggressiveness of NB cells through up-regulating HNF4α. A and C. colony formation assay indicated that transfection of sh-HNF4α or *HNF4α* restored the changes in cell growth of SGC-7901 and AGS cells induced by stable over-expression and knockdown of ITLN1, respectively. **B and D.** scratch assay indicated that transfection of sh-HNF4α or *HNF4α* restored the changes in migration of SGC-7901 and AGS cells induced by stable over-expression and knockdown of ITLN1, respectively.

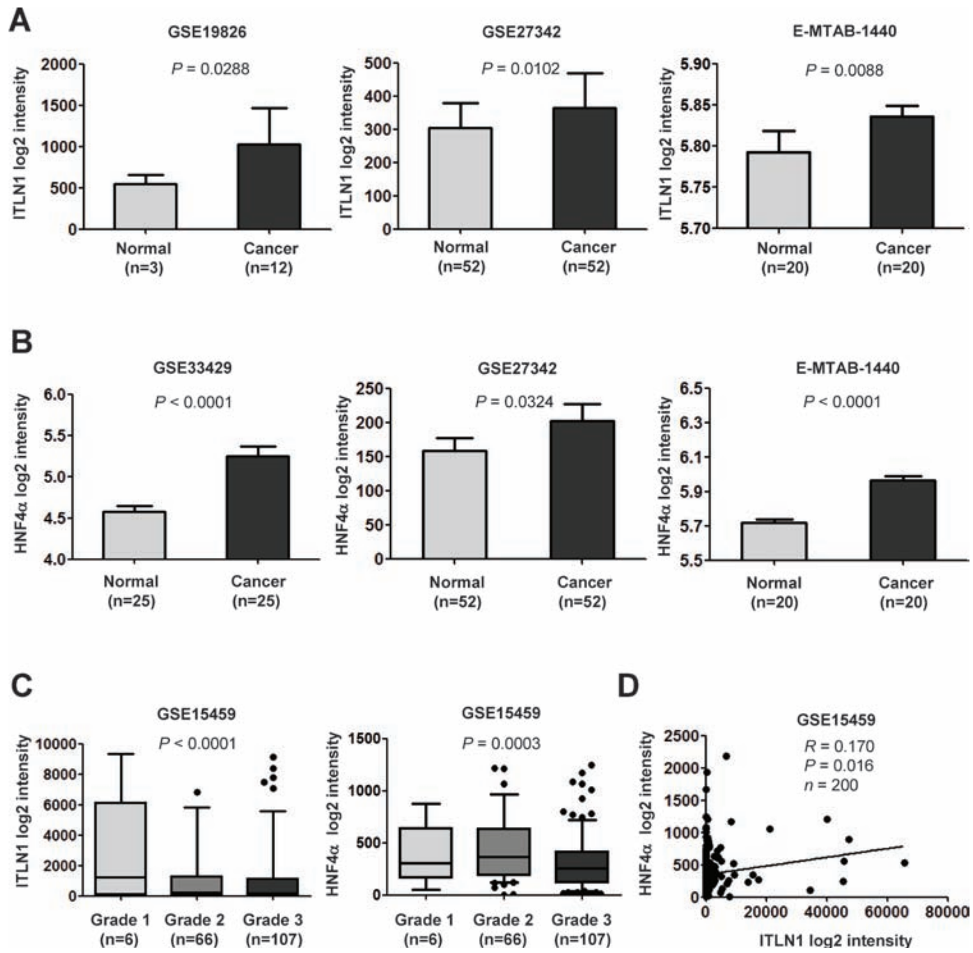

Supplementary Figure S5: Expression of ITLN1 and HNF4α in public databases. A. the ITLN1 levels were higher in gastric cancer tissues than those in normal gastric tissues in there independent cohorts derived from GEO datasets (http://www.ncbi.nlm.nih.gov/gds/) and arrayexpress database (http://www.ebi.ac.uk/arrayexpress/). **B.** the HNF4α levels were higher in gastric cancer tissues than those in normal gastric tissues in there independent cohorts derived from GEO datasets and arrayexpress database. **C.** the expression of ITLN1 and HNF4α was gradually decreased along with histological differentiation grades of gastric cancer tissues. **D.** there was a positive correlation between *ITLN1* and *HNF4α* transcript levels in gastric cancer tissues.

Supplementary Table S1. ITLN1-regulated 331 genes involved in cellular process

Up-regulated genes		Down-regulated genes				
ADAM11	MED29	ADCYAP1R1	EDA	IL8RB	OLIG3	SRF
AEBP1	NAP1L1	ADD2	EGFLAM	ILKAP	OPRM1	SRPX
AKT3	NDRG2	AGFG2	EIF4E3	IRF4	OSR1	SSTR2
AVPR2	NLRP5	ALDH1A2	EN1	IRF5	OVOL1	SSTR4
BACH1	NPHS1	APLN	EPO	ISLR2	PAX3	SSX5
BCL11B	PAIP2B	ARHGAP24	ERMN	ITGB8	PAX5	STAMBP
C3orf58	PAX2	ARHGAP33	ESM1	ITPKB	PAX7	STAR
CALY	PDZK1	ARHGEF10	ESRP1	KCNA5	PDE7A	STAT2
CCL3	PGRMC2	ASB1	EYA1	KCNMA1	PDGFRA	TBX21
CCNE2	PHF12	ATF7	EYA2	KDM6B	PHIP	TBX5
CDC42BPA	PLEKHG6	AXIN2	F10	KIAA1804	PHTF1	TCF12
CDH15	PRDM2	BCL11A	F2	KLF15	PKD1L2	TFAP2E
CDKN2B	PTPRT	BIRC8	FAM126A	LHCGR	PKDREJ	TFAP4
CHN2	RAB28	BMP5	FAM175A	LIG4	PLG	THAP5
CIDEA	RHPN1	BMPR2	FES	LILRA3	POFUT1	TLE6
DERL3	RUNX2	BRSK1	FGF22	LILRB5	PREX2	TNFRSF13B
DIRAS3	S100B	C4orf31	FGF7	LOC729501	PRKCB	TNRC4
DNAJC16	SH3BP1	C8A	FGF8	LPAR3	PRKCG	TP53TG5
ELF1	SIGLEC15	CA8	FLT4	LPHN3	PTGER3	TRIOBP
ENY2	SIRPB1	CAMK1D	FNDC1	LRP12	PTHLH	TRPV4
EPHA3	SLC1A3	CAMK2D	FOXI1	LRRFIP1	PYGO1	TULP1
ERP44	SOX7	CAMTA1	FOXP1	LTB	PYY	UCMA
FAIM2	SRCAP	CAV3	FPR2	LZTS1	RAB3B	VGLL2
FAM132A	SSX1	CCKBR	FZD1	MAF	RAB6B	WFIKKN2
FKBP1B	SUN2	CCL13	GBP2	MAMSTR	RARA	WNK2
FZD4	SYNGAP1	CCND2	GFI1	MBD2	RASGRF1	WNT3A
FZD5	TAS2R16	CD180	GON4L	MCF2L2	RASIP1	WNT4
GABRA4	TGFA	CD86	GPM6B	MED28	RBM20	ZBTB40
GDNF	TNFSF4	CDS1	GPR17	MLL	REG1A	ZBTB49
GFI1B	TRAF1	CELF3	GPR37L1	MMP3	REG3A	ZNF169
GNA14	UNC84B	CHRDL1	GPR68	MPZL1	RORB	ZNF224
GNG2	WNT10A	CHRM5	GPR87	MRAP2	RPRM	ZNF273
GPR55	ZBTB16	CHRNA3	GREM1	MSTN	RSU1	ZNF407
GRIK4	ZMIZ1	CHRNB3	GRHL1	MYCL1	S100A9	ZNF471
GULP1	ZNF19	CLEC11A	GRIK2	MYH6	SAMSN1	ZNF484

(*Continued*)

Up-regulated genes		Down-regulated genes				
HIPK3	ZNF226	CPEB2	GRIN2A	MYT1	SCAI	ZNF497
HMGA2	ZNF296	CSF1	GRP	NCKIPSD	SCAND2	ZNF509
HNF4A	ZNF518B	CTSS	GSC2	NEUROD6	SEMA6A	ZNF516
IKZF2	ZNF613	CXCL5	HAND1	NEUROG2	SERPINB13	ZNF548
IL17RD	ZNF799	CXCR5	HCRTR2	NFATC2	SERPINF1	ZNF682
IL18RAP	ZNF812	CXXC4	HEMGN	NF-E4	SERPINI1	
IL28RA		CYSLTR2	HHIP	NHLH1	SERPINI2	
IL29		D4S234E	HOXC11	NKX3-2	SH2D3C	
ING3		DACT1	HSD17B12	NLGN3	SIPA1L3	
INPP5J		DAZL	HSPA2	NLRC3	SLC8A1	
ITGA2B		DCC	HTR2B	NOX1	SMAD9	
MADCAM1		DGKI	IGFBP2	NRP2	SNX26	
MAP1B		DKK2	IL18R1	NTRK2	SOX12	
MAP2K5		DLX2	IL20	NTRK3	SOX21	
MAPKSP1		DMRT3	IL22RA2	OCRL	SP100	

Supplementary Table S2. ITLN1 expression in human gastric cancer tissues

Clinicopathologic features	Total	High ITLN1 transcript levels	*P*-value
	n (%)	*n* (%)	
**Age (years)**			
≤60	32 (35.5)	15 (46.9)	1.000
>60	58 (64.5)	27 (46.5)	
**Gender**			
Male	62 (68.9)	29 (46.8)	1.000
Female	28 (31.1)	13 (46.4)	
**Size (diameter)**			
≤6 cm	46 (51.1)	22 (47.8)	0.836
>6 cm	44 (48.9)	20 (45.4)	
**Laurén classification**			
Intestinal type	65 (72.2)	30 (46.2)	1.000
Diffuse type	25 (27.8)	12 (48.0)	
**Histological grade**			
High differentiation	22 (24.5)	21 (95.5)	0.019
Median differentiation	30 (33.3)	17 (56.7)	
Low/Poor differentiation	38 (42.2)	4 (10.5)	
**Gastric wall invasion**			
T1/T2	11 (12.2)	11 (100.0)	<0.001
T3/T4	79 (87.8)	31 (39.2)	
**Lymph node metastasis**			
Negative	25 (27.8)	23 (92.0)	<0.001
Positive	65 (72.2)	19 (29.2)	
**TNM stage**			
I/II	16 (17.8)	14 (87.5)	0.001
III/IV	74 (82.2)	28 (37.8)	

ITLN1, intelectin 1; TNM, tumor-node-metastasis

Supplementary Table S3. Oligonucleotide sets used for constructs and short hairpin RNAs

Oligo Set	Sequences
pcDNA3.1-ITLN1	5′-CGCCCAAGCTTATGAACCAACTCAGCTTC-3′ (sense);<br>5′-CGCGGATCCTCAACGATAGAATAGAAGCAC-3′ (antisense)
pcDNA3.1-NFκB-p65	5′-CGCCCAAGCTTATGGACGAACTGTTCCCCCTC-3′ (sense);<br>5′-CCGCTCGAGTTAGGAGCTGATCTGACTCAGCAG-3′ (antisense)
pGL3-HNF4α	5′-CGGGGTACCCGGGGAATTGGAGGTGAATC-3′ (sense);
(-606/+128)	5′-CCGCTCGAGGGCCATGTCCATGTCGACGA-3′ (antisense)
pGL3-HNF4α	5′-AAACTGCGGGGGAACTGGAATTTAGCTCCCAGAA-3′ (sense);
(ΔNFκB)	5′-TTCCAGTTCCCCCGCAGTTTCCCGGTTCCC-3′ (antisense)
sh-Scb	5′-GAGGTAGCGTTTAGTACTTATCTCGAGATAAGTACTAAACGCTACCTC-3′ (sense);<br>5′-GAGGTAGCGTTTAGTACTTATCTCGAGATAAGTACTAAACGCTACCTC-3′ (antisense)
sh-ITLN1#1	5′-GATATGGAACTCATGTTGGTTCTCGAGAACCAACATGAGTTCCATATC-3′ (sense);<br>5′-GATATGGAACTCATGTTGGTTCTCGAGAACCAACATGAGTTCCATATC-3′ (antisense)
sh-wITLN1#2	5′-CCAGTGAAATATGGAGAAGGACTCGAGTCCTTCTCCATATTTCACTGG-3′ (sense);<br>5′-CCAGTGAAATATGGAGAAGGACTCGAGTCCTTCTCCATATTTCACTGG-3′ (antisense)
sh-HNF4α#1	5′-CCGGCCCCTACACCACCCTGGAATTCTCGAGAATTCCAGGGTGGTGTAGGTTTTTG-3′ (sense);<br>5′-AATTCAAAAACCCCTACACCACCCTGGAATTCTCGAGAATTCCAGGGTGGTGTAGG-3′ (antisense)
sh-HNF4α#2	5′-CCGGCGAGGTGTTGACGATGGGCAACTCGAGTTGCCCATCGTCAACACCTCGTTTTTG-3′ (sense);<br>5′-AATTCAAAAACGAGGTGTTGACGATGGGCAACTCGAGTTGCCCATCGTCAACACCTCG-3′ (antisense)
sh-NFκB-p65#1	5′-CCGGGCCTTAATAGTAGGGTAAGTTCTCGAGAACTTACCCTACTATTAAGGCTTTTTG-3′ (sense);<br>5′-AATTCAAAAAGCCTTAATAGTAGGGTAAGTTCTCGAGAACTTACCCTACTATTAAGGC-3′ (antisense)
sh-NFκB-p65#2	5′-CCGGCGGATTGAGGAGAAACGTAAACTCGAGTTTACGTTTCTCCTCAATCCGTTTTTG-3′ (sense);<br>5′-AATTCAAAAACGGATTGAGGAGAAACGTAAACTCGAGTTTACGTTTCTCCTCAATCCG-3′ (antisense)

ITLN1, intelectin 1; NFκB, NF-kappa B; HNF4α, hepatocyte nuclear factor 4 alpha; sh-Scb, scramble short hairpin RNAs.

Supplementary Table S4. Primer sets used for qPCR and ChIP

Primer set	Primers	Sequence	Product size (bp)	Application
ITLN1	Forward	5′-AATGGACCTGTTCTTCGT-3′	253	qPCR
	Reverse	5′-TCTGGGTAGACTGCTTTG-3′		
HNF4α	Forward	5′-CATGGACATGGCCGACTACAG-3′	122	qPCR
	Reverse	5′-CGTTGAGGTTGGTGCCTTCTGA-3′		
β-catenin	Forward	5′-TGCCAAGTGGGTGGTATAGAGG-3′	272	qPCR
	Reverse	5′-TCACGCAAAGGTGCATGATTT-3′		
AXIN2	Forward	5′-TCCATGACGGACAGCAGTGTAG-3′	130	qPCR
	Reverse	5′-GGGTTCTCGGGAAATGAGGTAG-3′		
CCND2	Forward	5′-ATTACCTGGACCGTTTCTTGGC-3′	160	qPCR
	Reverse	5′-TCCTGAGGCTTGATGGAGTTGT-3′		
RUNX2	Forward	5′-CGGAATGCCTCTGCTGTTATG-3′	113	qPCR
	Reverse	5′-TTTGTGAAGACGGTTATGGTCAAG-3′		
MMP3	Forward	5′-GATGGAGCTGCAAGGGGTGA-3′	263	qPCR
	Reverse	5′-TTCGGGATGCCAGGAAAGG-3′		
GAPDH	Forward	5′-AGAAGGCTGGGGCTCATTTG-3′	258	qPCR
	Reverse	5′-AGGGGCCATCCACAGTCTTC-3′		
HNF4α set 1	Forward	5′-CGGGGAATTGGAGGTGAATC-3′	198	ChIP
(-606/-409)	Reverse	5′-TTCTACCCTCAATGCTTTTGCA-3′		
HNF4α set 2	Forward	5′-TTAGAATGCCTGACTTGGGG-3′	182	ChIP
(-339/-158)	Reverse	5′-GATTTGGCTGTTTGTTGGTT-3′		

ITLN1, intelectin 1; HNF4α, hepatocyte nuclear factor 4 alpha; AXIN2, axin 2; CCND2, cyclin D2; RUNX2, runt-related transcription factor 2; MMP3, matrix metallopeptidase 3; GAPDH, glyceraldehyde 3-phosphate dehydrogenase
